# Differential allosteric modulation within dopamine D_2_R - neurotensin NTS1R and D_2_R - serotonin 5-HT_2A_R receptor complexes gives bias to intracellular calcium signalling

**DOI:** 10.1038/s41598-019-52540-8

**Published:** 2019-11-08

**Authors:** Michael Plach, Thorsten Schäfer, Dasiel Oscar Borroto-Escuela, Dorothée Weikert, Peter Gmeiner, Kjell Fuxe, Kristina Friedland

**Affiliations:** 1 Department of Chemistry and Pharmacy, Molecular and Clinical Pharmacy, Friedrich-Alexander-Universität Erlangen-Nürnberg (FAU), Erlangen, Germany; 2 Department of Neuroscience, Karolinska Institutet, Stockholm, Sweden; 3 Department of Chemistry and Pharmacy, Medicinal Chemistry, Friedrich-Alexander-Universität Erlangen-Nürnberg (FAU), Erlangen, Germany; 4 Pharmacology and Toxicology, Institute of Pharmacy and Biochemistry, Johannes-Gutenberg-Universität, Mainz, Germany

**Keywords:** Cellular neuroscience, Molecular neuroscience

## Abstract

Proceeding investigations of G protein-coupled receptor (GPCR) heterocomplexes have demonstrated that the dopamine D2 receptor (D_2_R), one of the hub receptors in the physiology of schizophrenia, interacts with both the neurotensin NTS1 (NTS1R) and the serotonin 5-HT_2A_ receptor (5-HT_2A_R) in cell lines and rodent brain tissue. *In situ* proximity ligation assay and BRET-based saturation experiments confirmed interacting receptor assemblies in HEK293T and neuronal HT22 cells. The NTS1R agonist NT(8-13) reduces the Gα_q_-mediated calcium signal in the NTS1R-D_2_R complex compared to the NTS1R monomer which could be reversed by D_2_R antagonists. The bivalent ligand CS148 (NTS1R-agonistic, D_2_R-antagonistic) increased the calcium response addressing the dimer, consistent with the effect of the monovalent ligands suggesting an allosteric D_2_R-mediated modulation. In contrast, the 5-HT_2A_R-D_2_R heteromer did not show a calcium-altering receptor-receptor interaction. Despite their common coupling-preference for Gα_q_, 5-HT_2A_R and NTS1R supposedly interact with D_2_R each in a unique mode. This remarkably diverse ligand-mediated signalling in two different D_2_R heteroreceptor complexes illustrates the complexity of receptor-receptor interactions and their potential of modifying cell responses to external stimuli. Therefore, GPCR heteromers may provide a very promising novel target for the therapy of neuropsychiatric disorders.

## Introduction

Neurotransmitters like neurotensin (NT), dopamine (DA) and serotonin (5-HT) act via G protein-coupled receptors (GPCRs), the largest family of pharmacologically relevant membrane receptors^1–6^. The understanding of pathophysiological processes involved in the development of neuropsychiatric disorders like depression, addiction, Parkinson’s disease or schizophrenia that are all closely connected to transmitter imbalances or receptor dysfunction, is vital for the improvement of established therapeutic approaches^7–12^. An increasing number of studies strengthens the theory that GPCRs do not only exist as monomers but can associate into functional homo- or heteroreceptor complexes with distinct pharmacological profiles^13,14^. Ligand binding, signal processing or receptor trafficking may be changed due to the formation of higher-order entities^15–18^.

High-resolution structures determined by x-ray crystallography of both the neurotensin receptor 1 (NTS1R) and the dopamine D_2_ receptor (D_2_R) have been described very recently^19–21^. These breakthroughs help to further understand their pharmacology in the central nervous system. The localization of NTS1R on neurons of dopaminergic circuits (e.g. nigrostriatal or mesocorticolimbic pathways) provide evidence of anatomical and functional association of the receptors of interest in brain areas closely linked to the pathophysiology of schizophrenia^22^. There, NTS1R and D_2_R substantially contribute to neuronal transmission and are very likely to modulate each other via allosteric receptor-receptor interactions^23,24^. Recent efforts tried to elucidate the formation of dimers consisting of NTS1R and/or D_2_R. Whereas within a NTS1R dimer, a dynamic model with interconverting interfaces was postulated, a bioinformatics approach has identified amino acid triplets likely to be involved in the physical NTS1R-D_2_R interaction^25–27^. Apart from that, the existence and activity of NTS1R-D_2_R heterodimers in cellular systems and rat brains has been investigated^28,29^. It has been shown by bioluminescence resonance energy transfer (BRET) and radioligand binding that the activated NTS1R is able to negatively modulate dopaminergic signalling via an immediate receptor-receptor crosstalk^30^. Further elucidation of the physiological distribution and functional properties promises more insight in their relevance as potential drug targets for the therapy of neurological disorders.

In addition to addressing dopamine receptors, current first-line atypical antipsychotics antagonize the serotonin receptor 2A (5-HT_2A_R)^31^. Having deduced sequential interface homologies between 5-HT_2A_R and D_2_R, the formation of heterodimers was demonstrated *in vitro*^32^. In this system, hallucinogenic 5-HT_2A_R agonists are likely to increase dopaminergic signalling while non-hallucinogenic agonists like 5-HT do not alter D_2_R-mediated effects^17,32,33^. Moreover, 5-HT_2A_R and D_2_R are co-existent in striatal regions making them interesting structures for further investigation regarding physical interaction, modulation and therapeutic relevance. It still needs to be clarified whether GPCR heteromers in general may be capable of transducing biochemical information in a notably different way from their corresponding monomeric entities. In the present study, we therefore investigate NTS1R-D_2_R and 5-HT_2A_R-D_2_R heterodimerization in transfected cells applying the *in situ* proximity ligation assay (PLA) and BRET between the protomers. Remarkably, the respective heteromers reveal different allosteric D_2_R-mediated modulation of the calcium response detected by live cell imaging, which emphasizes the complexity and uniqueness of receptor-receptor interactions.

## Results

### Neurotensin receptor 1 and dopamine receptor D2 form heteromers in HT22 cells and show characteristic signalling

**Figure 1:**
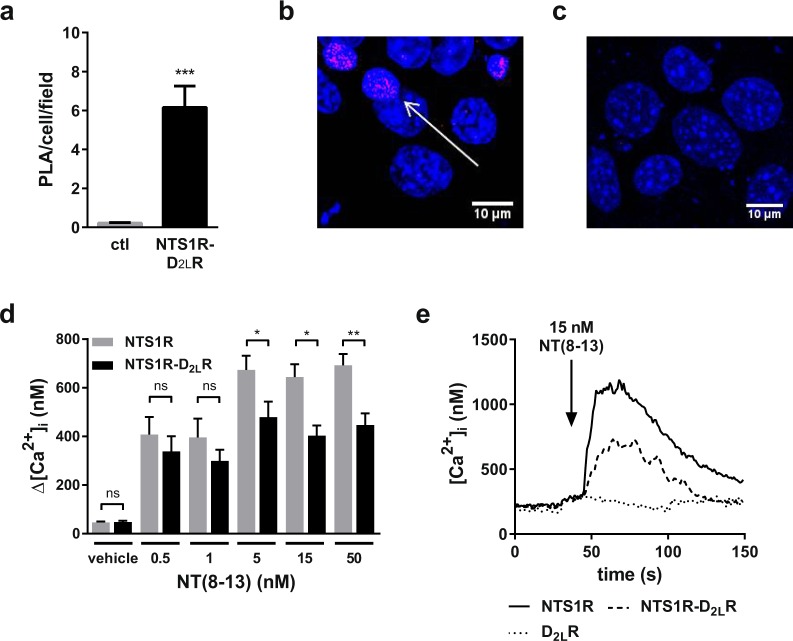
NTS1R-D_2_R heteromers show reduced calcium signalling. (**a**) NTS1R-D_2_R co-expressing HT22 cells showed high levels of PLA positive clusters as a marker for heteromeric receptor-receptor complexes, while only very few signals were detected in non-transfected cells (ctl) representing the non-specific background. Per sample field (150 µm × 150 µm), 52 cells in average were measured. (**b**) More than six PLA positive clusters (in pink, at tip of white arrow) per cell per sample field could be detected on average, which was significantly above background (***p < 0.001). (**c**) PLA signal is lacking in non-transfected cells. (**d**) Reduced intracellular Ca^2+^ responses in NTS1R-D_2_R co-expressing cells compared to NTS1R mono-expressing cells. Significant differences appear at NT(8-13) concentrations of 5 nM (Δ[Ca^2+^]_stimulated_ 194.7 ± 85.9 nM, *p < 0.05), 15 nM (Δ[Ca^2+^]_stimulated_ 241.4 ± 66.9 nM, *p < 0.05) and 50 nM (Δ[Ca^2+^]_stimulated_ 244.9 ± 67.1 nM, **p < 0.01). Values for vehicle treatment were deduced from Fura-2 ratio in HBSS before addition of ligand dilutions. (**e**) Imaging of immediate NTS1R mediated intracellular calcium release after application of 15 nM of NT(8-13) in transiently transfected HT22 cells (NTS1R: solid line, NTS1R-D_2L_R: dashed line, D_2L_R: dotted line). Shown curves represent mean response of 10–30 individual cells. Data were analyzed with student’s t-test and presented as mean ± SEM, n = 5–6, performed in hexaplicates.

As an initial step, we aimed to strengthen the hypothesis that neurotensin NTS1 and dopamine D_2_ receptors are able to form heteromers in a neuronal cell line. We applied the antibody based *in situ* PLA to visualize the heteromerization of NTS1R and D_2_R receptors in the plasma membrane of transiently transfected, fixed HT22 cells, an immortalized cell line of murine hippocampal origin^34^. Subsequent to a rolling circle amplification, the red fluorescent hybridization product that can only appear when the two relevant GPCRs are within a mutual distance of 10–20 nm was detected by confocal microscopy^35^. NTS1R-D_2_R co-transfected cells showed significantly high numbers of PLA positive clusters (Fig. 1a,b) indicating the formation of receptor heteromers. To show specificity, we added both primary NTS1R and D_2_R antibodies and the PLA required secondary antibodies to non-transfected cells. There, no statistically significant amount of PLA positive signals could be observed proving the selectivity of the method (Fig. 1a,c) and validating the appearance of heteromers in our cellular model.

After verifying the existence of D_2_R-NTS1R heteromers, we further examined how NTS1R Gα_q_-protein mediated calcium signalling changes with the impact of the partner receptor protomer expressed in close proximity. Activation of NTS1R by NT(8-13), the pharmacologically active part of its endogenous ligand neurotensin, initiates the signalling cascade mediated by phospholipase C that leads to a calcium release from the endoplasmic reticulum upon inositole trisphosphate binding^36^. This mechanism takes place within seconds, allowing the detection of immediate effects (Fig. 1e). The dose-response of NT(8-13) gives first evidence for altered calcium signalling in cells expressing only NTS1R or NTS1R-D_2_R heteromers (Fig. 1d). Upon stimulation with 5–50 nM NT(8-13), we observed a significantly decreased calcium signal in the co-expressing compared to NTS1R mono-expressing HT22 cells. Even NT(8-13) concentrations below 5 nM by trend led to reduced calcium levels in NTS1R-D_2_R cells (Fig. 1d,e). As indicated in Fig. 1e, NT(8-13) did not initiate any calcium changes in D_2_R mono-expressing cells and hence direct regulation by NT(8-13) interacting with D_2_R is very likely to be precluded. Thus, the D_2_R protomer *per se*, without agonist activation, seems to modulate the NT(8-13) mediated NTS1 receptor signalling, irrespective of excess of one of the receptors (Supplementary Fig. S1).

### Effects of D_2_R ligands on NTS1R-D_2_R mediated calcium signalling

**Figure 2:**
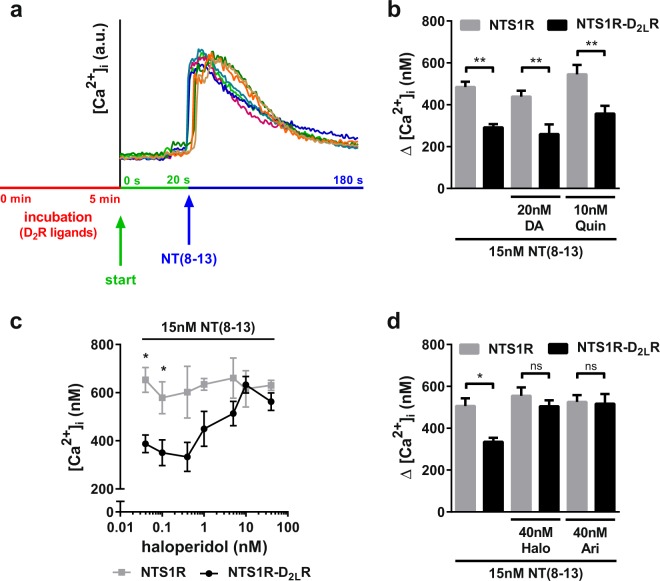
Dopamine receptor antagonists restore [Ca^2+^]_i_ response in NTS1R-D_2_R heteromers after stimulation with NT(8-13) in HT22 cells. (**a**) Experimental timeline of calcium imaging: 5 min incubation with dopaminergic ligands (red) is followed by washing steps before the start of the measurement. Application of NT(8-13) after 20 s (blue arrow) leads to NTS1R-mediated calcium release. End of recording after 3 min. Each colored curve represents one single cell. (**b**) Incubation with the D_2_R agonists dopamine (Δ[Ca^2+^]_stimulated_ 179 ± 47.5 nM) and quinpirole (Δ[Ca^2+^]_stimulated_ 188 ± 49.9 nM) did neither compensate nor enhance reduced Ca^2+^ levels in NTS1R-D_2_R co-expressing cells still showing significant differences towards the mono-transfected system (**p < 0.01). (**c**) In contrast, increasing concentrations of the dopaminergic antagonist haloperidol led to similarly high intracellular Ca^2+^ compared to NTS1R-only HT22 cells. (**d**) Incubation with 40 nM haloperidol or 40 nM of the weak D_2_R partial agonist aripiprazole led to a full recovery of the intracellular Ca^2+^ response in co-expressing cells after stimulation with 15 nM NT(8-13) no longer presenting significant differences between both expressing systems (*p < 0.05, ns = non-significant). Data were analyzed with one-way ANOVA and Tukey’s multiple comparisons test presented as mean ± SEM, n = 6.

Next, we investigated if D_2_R agonists can regulate the observed negative modulation within the NTS1R-D_2_R heterodimer. Therefore, the cells were incubated with dopamine and quinpirole, respectively, for 5 min before stimulation with 15 nM NT(8-13) (Fig. 2a), a concentration sufficient to evoke a maximum calcium response (Fig. 1d). Neither dopamine nor quinpirole were able to restore or intensify the calcium release provoked by NT(8-13) in NTS1R-D_2_R expressing cells (Fig. 2b). Co-transfected HT22 cells still showed approximately 35% lower intracellular calcium levels after activation compared to NTS1R mono-expressing cells, indicating that the intrinsic dopaminergic modulation is already at its maximum efficacy in the basal receptor state.

Subsequently, the effect of D_2_R antagonists on the antagonistic allosteric NTS1R-D_2_R interaction was analyzed. NTS1R mono-transfected cells were not affected by incubation with the D_2_R antagonist haloperidol, leading to a plateau calcium concentration of 624 ± 30.2 nM, mean ± SEM (Fig. 2c). In co-expressing cells, subnanomolar and low nanomolar concentrations of haloperidol did not change the allosteric receptor-receptor interaction exerted by the D_2_R protomer on the partner NTS1R protomer signalling. However, increasing concentrations of haloperidol (10–50 nM) resulted in intracellular calcium levels approaching the level of NTS1R mono-transfected cells. The weak partial D_2_R-like agonist aripiprazole showed a similar effect on the calcium signalling in NTS1R-D_2_R expressing cells (Fig. 2d). Since haloperidol and aripiprazole were able to block the constitutive D_2_R-mediated allosteric modulation within the NTS1R-D_2_R complex, we conclude that by antagonizing the D_2_R protomer the heterodimeric complex might change insofar as the inhibitory allosteric receptor-receptor interaction can no longer occur, thus leading to the same “full” calcium response in NTS1R-D_2_R heteromers as in NTS1R monomers.

**Figure 3:**
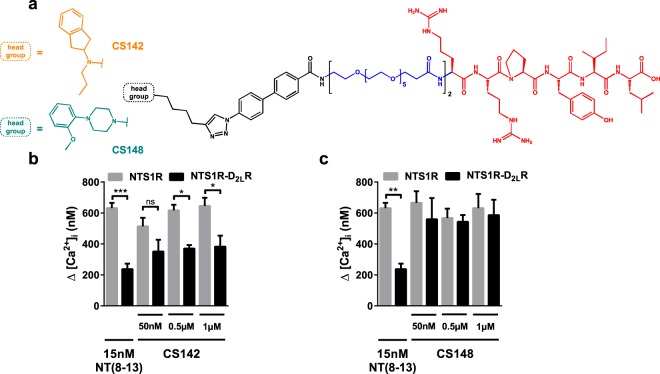
NTS1R-D_2_R bivalent ligand induced calcium release in HT22 cells. (**a**) The applied bivalent ligands consist of three distinct structural elements: NTS1R-agonistic pharmacophore NT(8-13) (red), spacer (blue) and D_2_R-agonistic (CS142, black-orange) or D_2_R-antagonistic (CS148, black-cyan) pharmacophore, respectively. (**b**) Treatment with 0.5–1 µM CS142 resulted in significantly reduced intracellular Ca^2+^ fluxes in co-expressing compared to NTS1R singly expressing cells (*p < 0.05). (**c**) The application of CS148 neutralized the diminished Ca^2+^ release in the NTS1R-D_2_R co-expressing system. The observed calcium recovery is displayed by non-significant differences between mono- and co-transfected cells for all tested concentrations. Data were analyzed with one-way ANOVA and Tukey’s multiple comparisons test presented as mean ± SEM, n = 6, performed in hexaplicates. *p < 0.05, **p < 0.01, ***p < 0.001, ns = non-significant.

To further support the D_2_R-mediated allosteric effect on the NTS1R dependent release of intracellular calcium, two recently described heterobivalent ligands, CS142 and CS148^37^, were applied to co-expressing HT22 cells. In contrast to the prior used substances that solely address the orthosteric binding site of either of the two receptors, these bivalent ligands comprise two independent pharmacologically active head groups that are connected by an appropriate spacer. Therefore, such compounds serve as perfect tools to co-stimulate closely adjacent receptors stabilizing physically interacting protomers. Both ligands contain the NTS1R agonistic pharmacophore NT(8-13). CS142 features a D_2_R agonistic aminoindane partial structure, whereas CS148 incorporates a 1,4-disubstituted-piperazine head group exhibiting antagonistic properties at the D_2_R protomer (Fig. 3a)^37^. We expected the simultaneous binding to provide further evidence of calcium signalling of NTS1R-D_2_R heteromeric origin. Consistent with the results obtained with monovalent dopamine receptor agonists, the inhibitory effect of the D_2_R protomer in the co-expressing cell system remained untouched by the D_2_R-agonistic bivalent ligand CS142. Consequently, the calcium level is significantly lower in comparison to HT22 cells that express NTS1R only (Fig. 3b). Stimulation with CS148, consisting of NT(8-13) and a D_2_R antagonist, resulted in calcium concentrations that are highly comparable to NTS1R mono-expressing cells (Fig. 3c). These observations support the conclusion that supposedly an allosteric inhibitory mechanism within NTS1R-D_2_R heteromers regarding G protein-mediated downstream signalling is present. Again, dopaminergic agonism does not affect this modulation, yet D_2_R-antagonism seems to rescue decreased intracellular calcium levels.

### Unchanged calcium signal in co-expressed 5-HT_2A_ and D_2_ receptors

**Figure 4:**
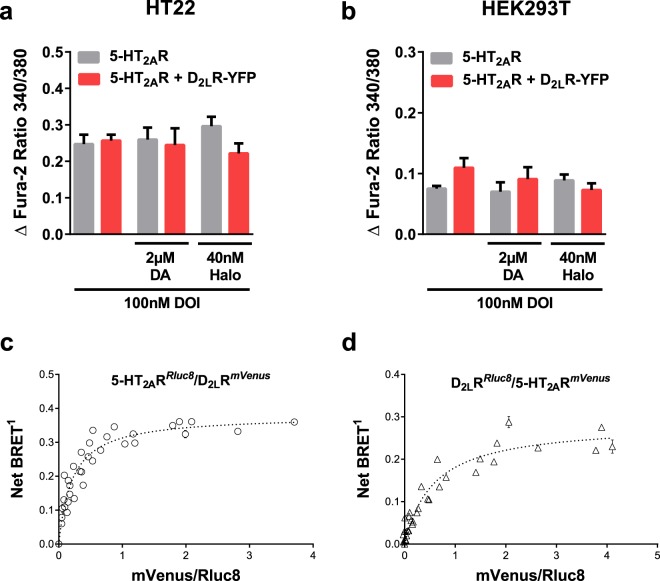
Ca^2+^ release following 5-HT_2A_R activation and BRET saturation assay to confirm specific interactions. Neither in (**a**) HT22 nor in (**b**) HEK293T quantitative differences in intracellular calcium could be detected in co-expressing cells upon stimulation with the 5-HT_2A_R agonist DOI. The amount of released calcium is depicted as a ratio of Fura-2, excited at 340 nm in the calcium bound and at 380 nm in the unbound state. Activation or inibition of the D_2_R protomer did not alter [Ca^2+^]_i_. For each treatment, all differences between 5-HT_2A_R and 5-HT_2A_R-D_2_R cells were non-significant. Data were analyzed with one-way ANOVA and Tukey’s multiple comparisons test presented as mean ± SEM, n = 6, performed in hexaplicates. (**c**) Saturated BRET titration curve for increasing concentrations of D_2L_R-mVenus as BRET acceptor and constant amounts of 5-HT_2A_R-Rluc8 as a donor. Close proximity of donor and acceptor molecule exerts in energy transfer after enzymatic conversion of the substrate coelenterazine-h indicating a direct interaction between both receptors. (**d**) Comparable positive BRET signals detectable for swapped donor-acceptor pair. Pooled data, performed in HEK293T cells, presented as mean ± SEM, n = 3.

As a next step, we investigated whether the D_2_R-based effects on the ligand-triggered calcium release are specific for the NTS1R-D_2_R heterodimer. Therefore, we changed the Gα_q_-coupled protomer and co-expressed the D_2_R together with the serotonin 5-HT_2A_R. Co-localization as well as direct interaction of D_2_R and 5-HT_2A_R have previously been described in HEK293 cells and in rat brain tissue^32,33^. Especially regions in the dorsal striatum and the nucleus accumbens are rich in 5-HT_2A_R-D_2_R complexes^33^. Again, HT22 cells served as the experimental model and expressed the 5-HT_2A_R either alone or together with the D_2_R after transfection. Interestingly, activation of the 5-HT_2A_R by its selective agonist DOI (100 nM) or its endogenous ligand 5-HT did not result in altered calcium levels independent of the presence of the D_2_R (Fig. 4a, Supplementary Figs S2 and S3). Varying the ratio of co-transfected cDNA did not significantly change the quality of the signal (Supplementary Fig. S4). Neither agonism by high concentrations of dopamine (2 µM) nor antagonism at the D_2_R binding site by haloperidol (40 nM) before DOI stimulation were able to affect the amount of calcium released intracellularly. These findings clearly differ from the allosteric interaction detectable in the NTS1R-D_2_R heterodimer. To exclude that these observations are cell line specific, calcium imaging was additionally performed in HEK293T cells. Again, we did not detect quantitative changes in the concentration of released calcium in mono- or co-expressing cells, with or without preincubation with dopaminergic ligands (Fig. 4b). In conclusion, we assume that independent of the *in vitro* test system, 5-HT_2A_R and D_2_R do not show a significant positive or negative receptor-receptor modulation, at least at the level of downstream calcium signalling. Hence, the NTS1R-D_2_R heterodimer allosteric interaction is a highly specific means of regulation.

To exclude that the absence of allosteric modulation does not rely on the absence of 5-HT_2A_R-D_2_R heteromers, we analyzed whether 5-HT_2A_R and D_2_R are expressed in immediate proximity, thus theoretically enabling a mutual physical interaction. To this end, we made use of a BRET saturation assay. Either of the receptors was C-terminally tagged with a coelenterazine-converting enzyme *Renilla reniformis* luciferase variant (Rluc8), whereas the other protomer served as the acceptor bearing the yellow fluorescent protein mVenus at its intracellular C-terminus. Keeping the concentration of the Rluc8 donor construct constant, we transfected HEK293T cells with increasing amounts of mVenus-fused receptor plasmids. Irrespective of the orientation of the BRET pair, characteristic saturable BRET titration curves could be obtained (Fig. 4c,d) indicating a specific protein-protein interaction^32,38^. This supports the assumption that the absence of any allosteric modulation regarding the calcium signalling does not depend on the absence of heterodimers.

## Discussion

There is evidence that heteromeric GPCR complexes differ in their way of transferring external stimuli to the inside of a cell compared to their monomeric counterparts. For instance, the activation of the dopamine receptor heterodimer D_1_R-D_2_R results in an unexpected Gα_q_-mediated calcium release, whereas the individual monomers modulate the effector adenylyl cyclase in response to ligand binding^39^. This ‘non-rigidity’ of interacting GPCRs can multiply the possibilities of signal transduction opening up a wide field for GPCR-targeting novel therapeutics.

In the present study, we demonstrate that two different D_2_R-containing GPCR heterocomplexes, each with a Gα_q_-coupling partner (NTS1R and 5-HT_2A_R, respectively), do not show identical behavior determining the intracellular calcium release after ligand activation. Immortalized neuronal HT22 cells served as a relevant model to investigate schizophrenia-related receptor interactions^34^. Interestingly, according to our data the mere co-expression of NTS1R and D_2_R is sufficient to reduce the level of intracellularly released calcium after NTS1R activation with NT(8-13) compared to singly expressed NTS1R. Being aware that the reduced signal may be influenced by the degree of receptor expression in the cells, we transfected different cDNA ratios. In each proportion, a significantly lowered dimer-specific signal could be obtained (Supplementary Fig. S1). The application of dopaminergic ligands provided more information about this negative allosteric modulation. We presume that blocking the D_2_R leads to a conformational change in the transmembrane helices involved in the NTS1R-D_2_R interface. This altered inter-receptor contact may change the geometrical properties of the NTS1R, which could result in an improved recruitment of Gα_q_ and therefore in a calcium signal identical to the monomeric response. In contrast, co-activation of the heterodimer with NT(8-13) and DA or quinpirole, respectively, does not seem to contribute to a relevant change in the interacting receptor domains indicated by the reduced intracellular calcium response. The application of bivalent ligands, which prevented the protomers from dissociation by their high-affinity bivalent binding mode^37^, supported that the detectable effect is dimer-specific and not provoked by disruption of the heterodimer. We suggest the notion of allosteric interaction via immediate conformational changes within the GPCRs, since the phospholipase C-β mediated release of calcium from cytosolic stores upon ligand binding is a short-term effect^40^. Whereas we here describe a negative receptor modulation arising from the D_2_R protomer towards the NTS1R, Borroto-Escuela *et al*. revealed, on the contrary, a direct receptor-receptor interaction within the NTS1R-D_2_R heterodimer^27^. Stimulation of the NTS1R led to an antagonistic modulation of the D_2_R-mediated adenylyl cyclase inhibition. On the other hand, they postulate that co-stimulation of both protomers accounts for a synergistic activation of the protein kinase C, likely involving agonist-mediated β-arrestin recruitment, resulting in an enhanced D_2_R signalling. Moreover, bivalent ligands with high affinity to NTS1R-D_2_R heteromers show a characteristic signalling behavior regarding cAMP accumulation in co-expressing compared to D_2_R singly expressing cells, potentially implying allosteric modulatory mechanisms^37^. Consequently, it becomes evident that allosteric receptor-receptor interactions by no means are clearly directional but are able to appear within one GPCR complex both positively and negatively in either direction.

Surprisingly, we did not see any influence on the intracellular calcium response in 5-HT_2A_R-D_2_R complexes compared to the mono-expressed 5-HT_2A_R, irrespective of the 5-HT_2A_R-D_2_R ratio (Supplementary Fig. S4). Neither any constitutive, nor any D_2_R agonist- or antagonist-mediated allosteric interaction was detected. This, however, does not automatically demonstrate the absence of any direct crosstalk, but at least at the level of downstream calcium signalling there appears to be a lack of allosteric modulation. Nevertheless, 5-HT_2A_R-D_2_R interactions have been described, mainly investigating D_2_R-promoted signalling^32,33^. Depending on the ability of the 5-HT_2A_R agonist to provoke hallucinations or not, the D_2_R signalling was influenced differently. Hallucinogenic DOI enhanced, but non-hallucinogenic 5-HT_2A_R ligands on the contrary rather diminished the Gα_i_-mediated actions of the D_2_R protomer. The authors concluded to have witnessed a dynamic set of allosteric receptor-receptor interactions that develop through conformational changes at the receptor interface upon ligand binding^33^. A similar bias was apparent at the level of inositol phosphate production, a process upstream of Ca^2+^-release from intracellular stores, after 5-HT_2A_R activation with and without D_2_R co-expressed or activated, respectively^17^. In the latter study, dimeric modulation was ligand and readout dependent. Contrarily, there is evidence for increased calcium signalling after 5-HT_2A_R-D_2_R co-stimulation^32^. There, the ensemble cell assay depicting combined signals of mono- and co-expressing cells may account for the signal enhancement in contrast to our single cell imaging technique where exclusively 5-HT_2A_R-D_2_R cells were analyzed. By trend, this finding might still be reflected in our HEK293T cell system showing a slightly higher, but non-significant signal for the DOI/DA co-activation (Fig. 4b). Yet, in hippocampus derived HT22 cells, an altered calcium signal could not be detected. Occurrence of a different dimer-specific behaviour in a more neuron-like system possibly gives a hint that modulatory receptor-receptor interactions may be dynamic in the environment they appear. Thus, the mechanism of atypical antipsychotic drugs may also proceed via heteromeric complexes in various areas of the brain. This fact makes GPCR heterodimers to very exciting target structures for drug development that need to be studied further to gain more knowledge about their pharmacology.

Yet, there is still limited understanding of the structural determinants of the different signalling behaviour of these two pairs of receptors. To elucidate NTS1R-D_2_R interaction, Hübner *et al*. combined X-ray crystal structures and homology modelling to design a heterodimer model^37^. They suggested transmembrane helices (TM) 1, 2 and helix 8 to be involved in the formation of the interface, supported by binding data of heterobivalent ligands. Further attempts to analyze the interface between D_2_R and NTS1R or 5-HT_2A_R, respectively, have been made by bioinformatical approaches, where short amino acid sequences that are predominantly found in heteromeric complexes were identified^27,32^. According to these findings, NTS1R and D_2_R are likely to have triplets in common that interact via TMs 2, 4 and 6 as well as intracellular loop 3^27^. 5-HT_2A_R and D_2_R, contrarily, are described to share homologies in TM1 and TM3^32^. The motifs within NTS1R-D_2_R and 5-HT_2A_R-D_2_R interfaces are non-identical so that dimer-specific interactions might be possible. For the adenosine A2A receptor (A2AR)-D_2_R dimer, however, interdisciplinary approaches have unravelled TM4 and TM5 of D_2_R to form the potential interface^41^, strengthening the evidence that D_2_R is able to develop different interaction sites in different receptor complexes. Since NTS1R homodimerization has been described in a “rolling interface” model^25^, according to which the interacting TMs are interconverting, presence of NTS1R within a heterocomplex might result in dynamic changes at the interface in contrast to other possibly more rigid dimers. Postulating that allosteric modulation may be transduced via conformational changes, different adjacent structural elements at dimer interfaces would therefore contribute to the differences in ligand mediated calcium response for NTS1R-D_2_R compared to 5-HT_2A_R-D_2_R.

In the plasma membrane of neurons, 5-HT_2A_R and NTS1R are by far not the only receptors to interact with D_2_R. For instance, A2AR has been described to form heteromers with D_2_R showing a strong antagonistic relation. Activation of the A2AR decreases Gα_i_-mediated signalling of the D_2_R while promoting the recruitment of β-arrestin2 to the dopamine D_2_ protomer at the same time^42,43^. Interestingly, three different D_2_R-heterocomplexes show clearly individual and unique allosteric modulation illustrating the importance of potential receptor-receptor interactions for the neuronal system to adapt its signal transduction to physiologically altered environments.

We are aware that the neuronal cell line used does not perfectly represent the molecular environment of brain tissue. Within a synapse, the release, the local distribution and concentration as well as the reuptake of neurotransmitters are tightly controlled by regulatory mechanisms^44^. Nevertheless, the hippocampus-derived cells are an adequate model to study receptor-receptor interactions relevant to neuropsychiatric disorders.

**Figure 5:**
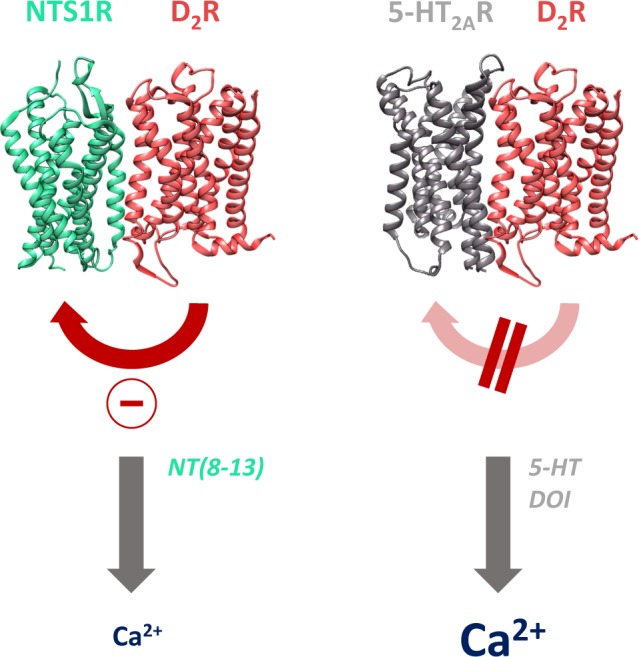
Interaction within heterogeneous receptor-receptor complexes. Differential allosteric modulation of the D_2_R protomer: Reduction of NTS1R-mediated calcium release via constitutive dopaminergic inhibition that is abolished in the presence of D_2_R antagonists. No positive or negative allosteric receptor-receptor interaction is observed for 5-HT_2A_R-D_2_R heterodimers, leading to a similar calcium response as detected for 5-HT_2A_R monomers. Receptor structures were taken from the Protein Data Bank and slightly adapted (PDB IDs: 4GRV (NTS1R), 6CM4 (D_2_R), 6A93 (5-HT_2A_R)).

In summary, we here demonstrate the existence of two different pairs of GPCR heteromers, namely the NTS1R-D_2_R and the 5-HT_2A_R-D_2_R using *in situ* PLA and BRET. There is evidence that the allosteric interactions between the protomers may be due to conformational anomalies or changes at facing transmembraneous domains. In addition, the extent of the inter-receptor modulation is highly dimer and ligand specific as we could show the altered modulation of intracellular calcium responses by live cell calcium imaging (Fig. 5). Having in mind that these heteromers are *inter alia* present in schizophrenia associated limbic areas^14^, it is of particular interest to continue the studies on dimer expression, distribution, monomer-dimer equilibrium and signalling. Further work is inevitably necessary to better understand these receptor formations in order to use them as potential targets for specific and effective therapy of neuronal diseases.

## Materials and Methods

### Reagents

Neurotensin 8–13 (NT(8-13)) and the bivalent ligands CS142 and CS148 were synthesized as previously described^37^. Dopamine, quinpirole and DOI were purchased from Sigma-Aldrich (Munich, Germany). NT(8-13), CS142, CS148, dopamine, quinpirole and 2,5-dimethoxy-4-iodoamphetamine (DOI) were used as aqueous dilutions. Stock solutions of haloperidol and aripiprazole were prepared in DMSO and diluted immediately before use.

### Plasmids

For calcium live imaging, wildtype human NTS1R, D_2L_R and 5-HT_2A_R in pcDNA3.1 (+) (cDNA Resource Center, www.cdna.org) and plasmids containing the cDNA for the fluorescently labeled receptor fusion proteins NTS1R-eCFP and D_2L_R-eYFP in pcDNA3.1 (+)^30^ were used. The cDNA encoding human 3xHA 5-HT_2A_R was designed in the lab of Kjell Fuxe, using standard molecular cloning. The BRET^1^ biosensors 3xHA 5-HT_2A_R-Rluc8, 3xHA 5-HT_2A_R-mVenus, D_2L_R-Rluc8 and D_2L_R-mVenus in pcDNA3.1 (+) were cloned applying the Gibson Assembly Cloning Kit (New England Biolabs, Frankfurt am Main, Germany) in accordance to the manufacturer’s protocol. All oligonucleotides were obtained from biomers.net (Ulm, Germany). Jonathan Javitch (Columbia University, New York, USA) provided the Rluc8 coding cDNA. The D_2L_R and 3xHA 5-HT_2A_R Rluc8 fusions were designed according to the previously described D_2S_R-Rluc8 construct^45^. mVenus constructs were cloned analogously. Hence, the cDNAs containing Rluc8 were fused to the C-termini of 3xHA 5-HT_2A_R or D_2L_R by a 24 amino acid linker (ATGLRSRAQASNSAVDGTAGPVAT), while a 5 amino acid linker (GGGAS) was used for mVenus. All constructs were verified by sequencing (Eurofins Genomics, Köln or LGC Genomics, Berlin, Germany).

### Cell culture and transfection

HT22 cells were cultured in Dulbecco’s modified Eagle’s medium (DMEM) supplemented with 10% heat-inactivated fetal bovine serum (FBS, Invitrogen, Karlsruhe, Germany), 2 mM L-glutamine and 20 mM HEPES, 100 U/mL penicillin and 100 µg/mL streptomycin (Invitrogen) in 10 cm culture dishes at 37 °C in a humidified incubator containing 5% CO_2_. HEK293T cells were grown in DMEM/F-12 supplemented with 10% heat-inactivated FBS, 100 U/mL penicillin and 100 µg/mL streptomycin. Passaging of cells took place every 3–4 days with 0.25% trypsin (Invitrogen) for HT22 or without trypsin for HEK293T cells, at a confluency of 75–100%. For *in situ* PLA and calcium imaging, 1 × 10^5^ HT22 or 2 × 10^5^ HEK293T cells were seeded on Poly-L-lysine coated 20 mm glass coverslips (LaCon, Erbach, Germany) in serum-reduced media (2% FBS). After 24 h at 37 °C, cells were transfected using serum-free media and Lipofectamine LTX Reagent with PLUS Reagent (Invitrogen) at a 3:1 transfection reagent to cDNA ratio. Per coverslip and receptor, 1 µg of cDNA was used.

### *In situ* proximity ligation assay

48 h after transfection, *in situ* PLA was performed as previously described^35^ using untransfected HT22 cells as controls. Subsequent to permeabilization and blocking, 4% PFA fixed cells were treated with rabbit polyclonal NTS1R antibody (1:500) (Abcam, Cambridge, UK) and mouse D_2_R antibody (1:600) (Merck, Darmstadt, Germany) in SuperBlock Blocking Buffer (Fisher Scientific, Schwerte, Germany) overnight at 4 °C. Thereafter, the proximity probe mixture consisting of 1:5 dilutions of Duolink *In Situ* PLA-Probe Anti-Rabbit PLUS and Anti-Mouse MINUS (Sigma Aldrich) in blocking buffer, respectively, was added (60 min, 37 °C) followed by ligation (ligase, 0.025 U/µL, 30 min, 37 °C) and rolling circle amplification (polymerase, 0.125 U/µL, 100 min, 37 °C, Duolink *In Situ* Detection Reagents Red, Sigma Aldrich). Nuclei were stained with Duolink Mounting Medium with DAPI (Sigma Aldrich). After drying, the coverslips were sealed with nail polish and stored at −20 °C. PLA signals were visualized using a Leica TCS-SL confocal microscope (Leica, Buffalo Grove, USA) equipped with a TexasRed filter (fluorophore emission at 624 nm) and quantified with Duolink Image Tool software (Olink, Uppsala, Sweden).

### Calcium live imaging

Single cell live imaging of [Ca^2+^]_i_ was performed using an ultra-fast switching monochromator (Polychrome V, FEI, Munich, Germany) with a xenon lamp (Tilluxe PX45, FEI) linked to a fluid immersion microscope (Olympus EX51WI, Hamburg, Germany) and the fluorescence indicator Fura-2-AM (Invitrogen). 48 h after mono-transfection with NTS1R or co-transfection with NTS1R and D_2L_R-YFP, HT22 or HEK293T cells were washed twice with HBSS (138 mM NaCl, 6 mM KCl, 1 mM MgCl_2_, 2 mM CaCl_2_, 5.5 mM glucose, 10 mM HEPES, pH 7.4, 37 °C). Experiments with 5-HT_2A_R alone or in combination with D_2L_R-YFP were performed 24 h after transfection, as low cell viability and frequent detachment were observed at later time points. Loading with Fura-2-AM, (0.35 µM in HBSS with 2% FBS, 0.01% Pluronic F-127 (Invitrogen)) was performed at 37 °C under light exclusion for 30 minutes. After two washing steps, cells were kept dark at room temperature for 30 minutes. Cells were incubated with either dopamine (20 nM, 2 µM), haloperidol (40 nM), quinpirole (10 nM), aripiprazole (40 nM) or HBSS (control) for 5 minutes at room temperature and transferred to the imaging chamber. Cells were alternately excited with 340 nm and 380 nm for 50 ms each. A 510 nm mirror unit was used to collect Fura-2 fluorescence. Following stimulation with NT(8-13), CS142 or CS148 as NTS1R agonists or 5-HT and DOI as 5-HT_2A_R ligands, the calculated 340/380 absorption ratio provided information about changes in intracellular calcium concentrations. Transformation of ratio values into absolute calcium concentrations was carried out according to literature^46^. Δ[Ca^2+^]_i_ values were determined calculating the difference between basal calcium before and maximum response after ligand application. D_2L_R transfected cells were identified using their eYFP fluorescent tag and only cells that showed eYFP fluorescence and concomitant release of calcium upon NTS1R or 5-HT_2A_R agonist application were analyzed for co-transfections. For the investigation of NTS1R or 5-HT_2A_R mono-transfected cells, only cells providing a calcium response were included in data analysis.

### BRET^1^ saturation assay

BRET saturation experiments have been described previously^47^. Briefly, HEK293T cells were transfected in suspension at a density of 1.25 × 10^5^ cells/mL in complete media using polyethylenimine (PEI, linear, 25 kDa, Polysciences, Heidelberg, Germany) at a 3:1 PEI to cDNA ratio. Increasing amounts of mVenus-labeled BRET acceptor cDNA (0–700 ng) were mixed with a constant amount of Rluc8-tagged donor cDNA (100 ng for 3xHA 5-HT_2A_R-Rluc8 and 50 ng for D_2L_R-Rluc8, respectively) and complemented with ssDNA (Sigma Aldrich) to a total amount of 1 µg cDNA per 1.2 mL cell suspension. The cDNA/PEI complexes were incubated at room temperature for 30 minutes, before the transfection complex was added to the cells and cells were transferred to white 96-well plates (100 µL/well, Greiner Frickenhausen, Germany). After 48 h at 37 °C and 5% CO_2_, the media was removed and substituted by 90 µL of PBS supplemented with CaCl_2_ and MgCl_2_ (Invitrogen) for 1 h. A CLARIOstar Plate Reader (BMG Labtech, Ortenberg, Germany) was used to determine mVenus fluorescence (excitation 497 nm, emission 535 nm). BRET^1^ measurements were performed 20 minutes after addition of 10 µL of coelenterazine-h (5 µM final concentration, Promega, Mannheim, Germany) using a BRET^1^ filter set (475 − 30/535 − 30 nm). The BRET^1^ signals were calculated as the ratio between the intensity of light emitted by the acceptor (mVenus, 535 nm) divided by the donor emission (Rluc8, 475 nm). Specific BRET (net BRET) was determined by subtraction of the signal obtained in the absence of any fluorescent acceptor protein and plotted against the ratio of fluorescence (mVenus) to luminescence (Rluc8) to receive saturation curves.

### Statistical analysis

Experimental data are presented as mean ± SEM. Graphical and statistical analysis was performed using GraphPad Prism 6.0 (GraphPad Software, Inc.; San Diego, CA, USA). One-way ANOVA or Student’s *t*-test together with post-hoc Bonferroni’s or Tukey’s test provide information about statistical significance. *p* values less than 0.05 were considered statistically significant (*p < 0.05; **p < 0.01; ***p < 0.001), above 0.05 as non-significant (ns). Number of single experiments ranges between n = 3–6.

## Supplementary information

Supplementary Information

## Data Availability

All data generated and analyzed during this study may be requested from the corresponding author (K.Fr.).
