# Supplementary material for: Differential allosteric modulation within dopamine D2R - neurotensin NTS1R and D2R - serotonin 5-HT2AR receptor complexes gives bias to intracellular calcium signalling
Source: Sci Rep. 2019 Nov 8;9:16312. doi: 10.1038/s41598-019-52540-8 (PMC6841725; doi:10.1038/s41598-019-52540-8)
Supplement: Supplementary file 1 — Supplementary Information [file 41598_2019_52540_MOESM1_ESM.pdf]

Differential allosteric modulation within dopamine D_2R - neurotensin NTS1R and D_2R - serotonin 5-$HT_{2A}R$ receptor complexes gives bias to intracellular calcium signalling

Michael Plach[1‡], Thorsten Schäfer[1‡], Dasiel Oscar Borroto-Escuela[2], Dorothée Weikert[3], Peter Gmeiner[3], Kjell Fuxe[2], Kristina Friedland[1,4]*

[1]Department of Chemistry and Pharmacy, Molecular and Clinical Pharmacy, Friedrich-Alexander-Universität Erlangen-Nürnberg (FAU), Erlangen, Germany

[2]Department of Neuroscience, Karolinska Institutet, Stockholm, Sweden

[3]Department of Chemistry and Pharmacy, Medicinal Chemistry, Friedrich-Alexander-Universität Erlangen-Nürnberg (FAU), Erlangen, Germany

[4]Department of Pharmacology and Toxicology, Institute of Pharmacy and Biochemistry, Johannes-Gutenberg-Universität, Mainz, Germany

‡ These authors contributed equally.

* Corresponding author: Kristina Friedland, Staudingerweg 5, 55128 Mainz, Germany, +49 6131 3924308, kfriedla@uni-mainz.de

Supplementary Figures

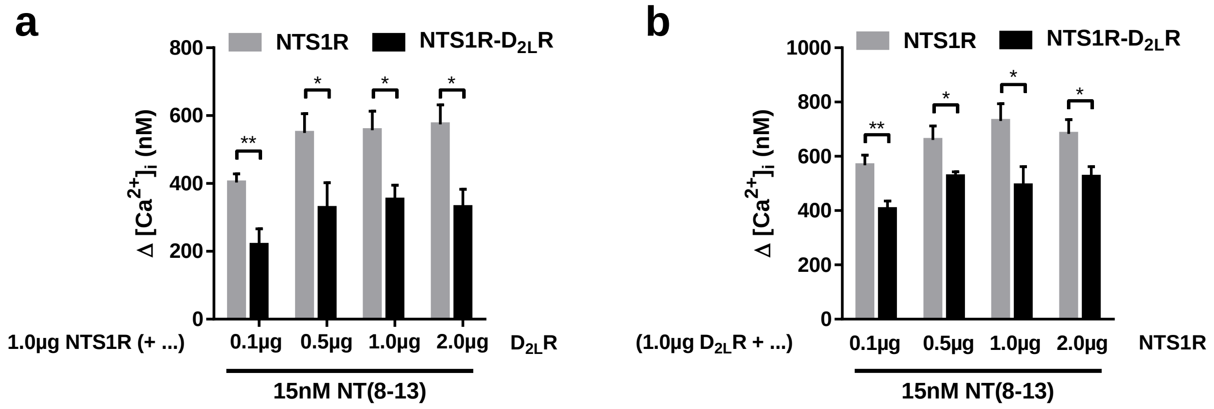

Supplementary Figure S1: Varying amounts of cDNA do not effect NTS1R-$D_{2L}R$ mediated intracellular calcium in HT22 cells. (a) NT(8-13) stimulation of HT22 cells, transiently co-transfected with 0.1 µg – 2.0 µg of $D_{2L}R$ cDNA and a constant amount of 1.0 µg NTS1R, led to a significantly lowered intracellular calcium response compared to NTS1R mono-expressing cells in all of the tested cDNA ratios. (b) Comparably, variation of NTS1R cDNA amounts resulted in markedly reduced calcium signals for co-expressing cells, covering a range between 0.1 µg and 2.0 µg for NTS1R and 1.0 µg for $D_{2L}R$. Data were analyzed with student's t-test and presented as mean ± SEM, n = 6, performed in hexaplicates. * $p < 0.05$, ** $p < 0.01$.

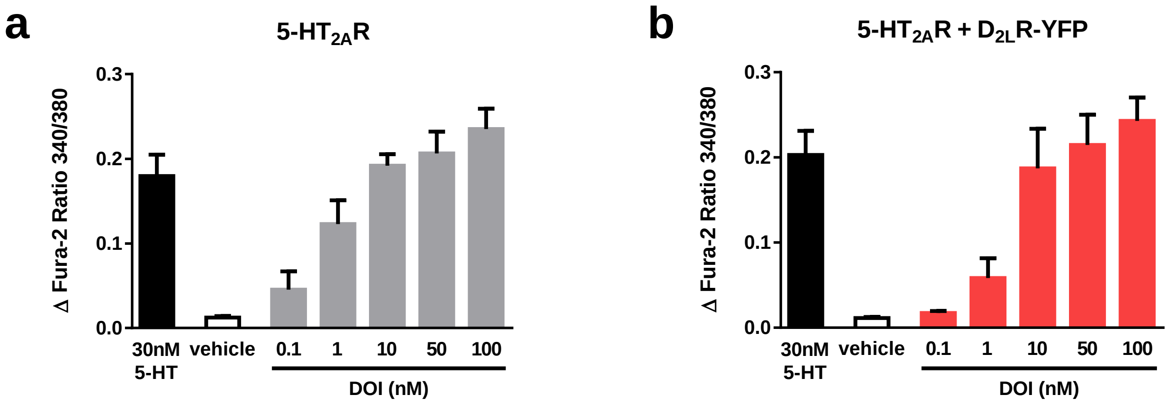

Supplementary Figure S2: Dose-response curves of DOI induced intracellular calcium in HT22 cells. (a) 5-$HT_{2A}R$ mono-transfected cells stimulated with 30 nM 5-HT as a positive reference, vehicle (HBSS) as a negative control and rising concentrations of 5-$HT_{2A}R$ agonist DOI (0.1 – 100 nM) led to a dose-dependent increase of ΔFura-2 ratio, representing the change in intracellularly released calcium. Concentrations above 10 nM approach a maximum response. (b) Cells co-expressing 5-$HT_{2A}R$ and D_2R showed a comparable dose-response profile with highly similar absolute values. Vehicle was deduced by ratio calculations before addition of the ligands.

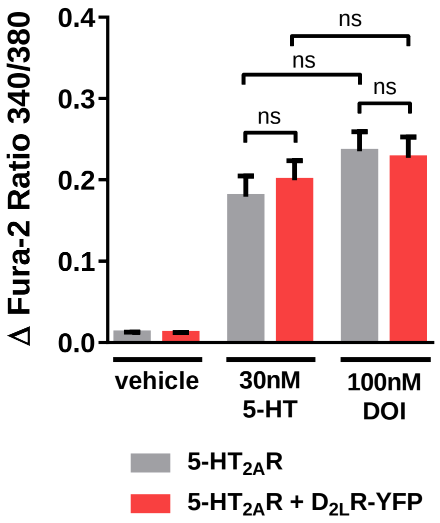

Supplementary Figure S3: 5-HT and DOI mediated calcium signals do not differ. Highly similar calcium release was obtained in mono- and co-expressing HT22 cells, independent of the 5-$HT_{2A}R$ activating ligand. Vehicle treatment (HBSS) was determined by calculation of the ratio before addition of ligands. Experiments were performed in triplicates, data analyzed with student's t-test and presented as mean ± SEM, n = 6-7, ns = non-significant.

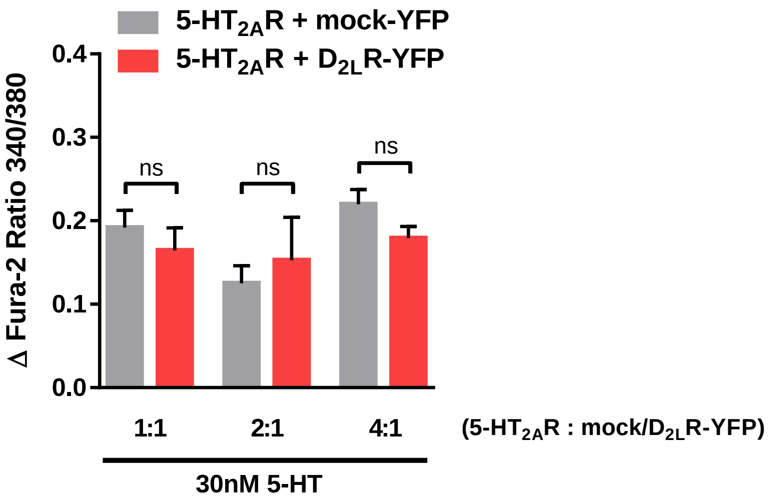

Supplementary Figure S4: Alteration of 5-$HT_{2A}R$/$D_{2L}R$ ratio does not change 5-HT provoked calcium response in HT22 cells. Neither equimolar amounts (1:1), nor 2- or 4-fold excess of 5-$HT_{2A}R$ towards $D_{2L}R$ or mock cDNA changed intracellular calcium signalling significantly ($p > 0.05$), since for all tested cDNA ratios responses were highly comparable for mono- and co-transfected cells. Data were analyzed with student's t-test and presented as mean ± SEM, n = 3-6, performed in hexaplicates, ns = non-significant.
